# From Adipose to Ailing Kidneys: The Role of Lipid Metabolism in Obesity-Related Chronic Kidney Disease

**DOI:** 10.3390/antiox13121540

**Published:** 2024-12-16

**Authors:** Wenchao Xu, Yuting Zhu, Siyuan Wang, Jihong Liu, Hao Li

**Affiliations:** 1 Department of Urology, Tongji Hospital, Tongji Medical College, Huazhong University of Science and Technology, Wuhan 430030, China; wcxutj@hust.edu.cn; 2 Institute of Urology, Tongji Hospital, Tongji Medical College, Huazhong University of Science and Technology, Wuhan 430030, China; 3 Department of Nephrology, Union Hospital, Tongji Medical College, Huazhong University of Science and Technology, Wuhan 430022, China; 4 Department of Geriatrics, Tongji Hospital, Tongji Medical College, Huazhong University of Science and Technology, Wuhan 430030, China

**Keywords:** obesity, chronic kidney disease, lipid metabolism, lipotoxicity, inflammation

## Abstract

Obesity has emerged as a significant public health crisis, closely linked to the pathogenesis and progression of chronic kidney disease (CKD). This review explores the intricate relationship between obesity-induced lipid metabolism disorders and renal health. We discuss how excessive free fatty acids (FFAs) lead to lipid accumulation in renal tissues, resulting in cellular lipotoxicity, oxidative stress, and inflammation, ultimately contributing to renal injury. Key molecular mechanisms, including the roles of transcriptional regulators like PPARs and SREBP-1, are examined for their implications in lipid metabolism dysregulation. The review also highlights the impact of glomerular and tubular lipid overload on kidney pathology, emphasizing the roles of podocytes and tubular cells in maintaining kidney function. Various therapeutic strategies targeting lipid metabolism, including pharmacological agents such as statins and SGLT2 inhibitors, as well as lifestyle modifications, are discussed for their potential to mitigate CKD progression in obese individuals. Future research directions are suggested to better understand the mechanisms linking lipid metabolism to kidney disease and to develop personalized therapeutic approaches. Ultimately, addressing obesity-related lipid metabolism disorders may enhance kidney health and improve outcomes for individuals suffering from CKD.

## 1. Introduction

### 1.1. Obesity as a Global Health Crisis

Obesity has increasingly emerged as a prominent public health crisis, with the World Health Organization (WHO) estimating that the prevalence of obesity has nearly tripled worldwide since 1975 [1]. In 2022, approximately 2 billion adults worldwide were classified as overweight, with 650 million (12% of the global adult population) categorized as obese. If current trends persist, projections estimate that by 2025, 2.7 billion adults will be overweight, and over 1 billion adults—representing 18% of men and 21% of women—will be obese [2]. This sharp rise underscores significant shifts in global lifestyle and dietary patterns. The epidemic of obesity is not confined to adults; it has also escalated among children and adolescents, raising concerns about long-term health implications [3,4]. This increase in obesity prevalence is closely linked to the rapid globalization of food systems, characterized by the widespread availability of energy-dense, nutrient-poor foods and reduced levels of physical activity due to sedentary lifestyles [5].

Obesity is associated with a myriad of chronic diseases, including cardiovascular diseases, type 2 diabetes, hypertension, and certain cancers [6,7,8,9,10]. Of particular concern is its relationship with chronic kidney disease (CKD) [11,12,13], a condition that has significant morbidity and mortality implications. Epidemiological studies indicate that individuals with obesity have an increased risk of developing CKD, and those who are already diagnosed with CKD often experience accelerated disease progression [14,15]. The mechanisms underlying the relationship between obesity and kidney health are complex and multifactorial [16,17]. They include metabolic dysregulation, chronic inflammation, and oxidative stress, all of which can lead to structural and functional alterations in the kidneys [18,19]. The pathophysiological processes linking obesity to CKD involve a cascade of events that disrupt normal renal function. Central to this relationship is the role of adipose tissue, which functions as an active endocrine organ, secreting a variety of bioactive molecules, including hormones and cytokines [20,21]. In the setting of obesity, there is a shift in the adipokine profile, with increased levels of pro-inflammatory leptin and decreased levels of protective adiponectin [22,23]. This dysregulation contributes to systemic inflammation, insulin resistance, and ultimately, renal injury [24,25,26]. Understanding the intricate connections between obesity and kidney health is crucial for the development of effective preventive and therapeutic strategies aimed at mitigating the impacts of obesity on renal function.

### 1.2. Role of Lipid Metabolism in Obesity-Related Kidney Disease

Lipid metabolism plays a critical role in maintaining energy homeostasis and cellular function within the kidneys [26,27]. The kidneys are essential for lipid metabolism, as they not only filter lipids from the bloodstream but also participate in lipid synthesis and oxidation. However, dysregulation of lipid metabolism in the context of obesity leads to excessive lipid accumulation in renal tissues, which is increasingly recognized as a primary mechanism driving renal injury in obese individuals [28,29]. The phenomenon of lipotoxicity—resulting from excessive lipid accumulation—can lead to cellular apoptosis, inflammation, and fibrosis, culminating in the progression of CKD [30,31]. Studies have shown that the accumulation of specific lipid species, such as free fatty acids (FFAs) and ceramides, can induce oxidative stress and mitochondrial dysfunction, further exacerbating renal damage [32,33]. In addition, the pro-inflammatory milieu created by excess lipids can activate various signaling pathways that promote renal fibrosis, glomerulosclerosis, and tubulointerstitial damage [34].

**Figure 1:**
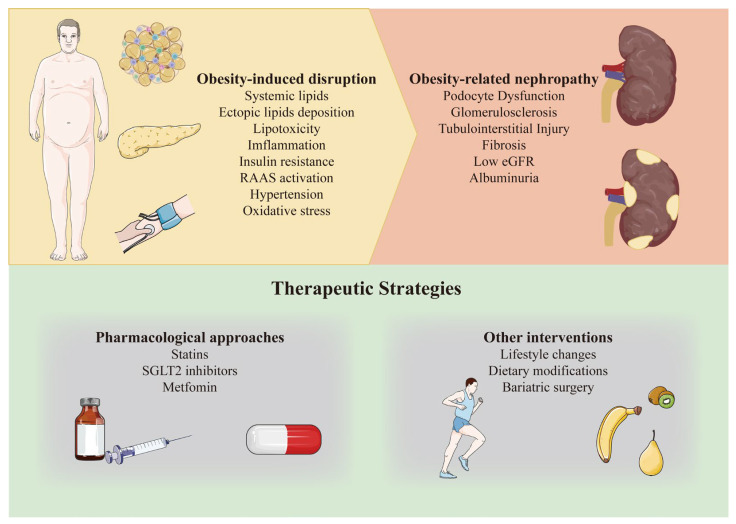
Pathophysiological Pathways and Therapeutic Strategies in Obesity-Related Nephropathy. Figure 1 illustrates both established and proposed obesity-induced mechanisms that contribute, directly or indirectly, to the development of obesity-related nephropathy. This condition is typically categorized by the presence and degree of albuminuria and the estimated glomerular filtration rate (eGFR), as well as by various forms of kidney-specific damage. The “Therapeutic Strategies” box highlights current and potential pharmaceutical and non-pharmaceutical approaches that target obesity and obesity-related nephropathy. RAAS, renin–angiotensin–aldosterone system; SGLT2, sodium–glucose cotransporter 2.

As the prevalence of obesity rises globally, the urgency to investigate lipid metabolism disorders and their implications for kidney health intensifies. An understanding of how obesity-induced alterations in lipid metabolism impact renal function is essential for developing targeted therapeutic strategies. This review aims to elucidate the mechanisms through which obesity-induced lipid metabolism disorders contribute to kidney dysfunction and to explore potential therapeutic targets for managing these conditions. By shedding light on these pathways, we hope to provide insights that will inform future research and clinical approaches to mitigate the adverse effects of obesity on kidney health (Figure 1).

## 2. Kidney-Specific Lipid Metabolism: Transporters, Lipogenesis, FAO

### 2.1. Lipid Metabolism Overview in the Kidney

The kidney plays a crucial and multifaceted role in lipid metabolism, contributing significantly to the uptake, processing, and utilization of various lipid classes [26,27]. The renal system has a unique lipid profile characterized by the presence of FFAs, triglycerides, phospholipids, and cholesterol [35]. These lipids are not only integral components of cell membranes but also serve as energy sources, signaling molecules, and modulators of inflammation [36]. Under physiological conditions, the kidney primarily utilizes FFAs as a significant energy substrate, particularly during periods of fasting or low carbohydrate intake [37]. The renal cortex, where most metabolic activities occur, is rich in mitochondria, reflecting its high energy demands [38]. In this context, the kidney’s ability to efficiently process and metabolize lipids is vital for maintaining overall renal function, energy homeostasis, and metabolic balance [39]. However, disturbances in lipid metabolism can lead to pathophysiological changes that predispose the kidney to various forms of injury [40], highlighting the need for a comprehensive understanding of the underlying mechanisms.

### 2.2. Lipid Uptake in Obesity-Related Kidney Disease

**Figure 2:**
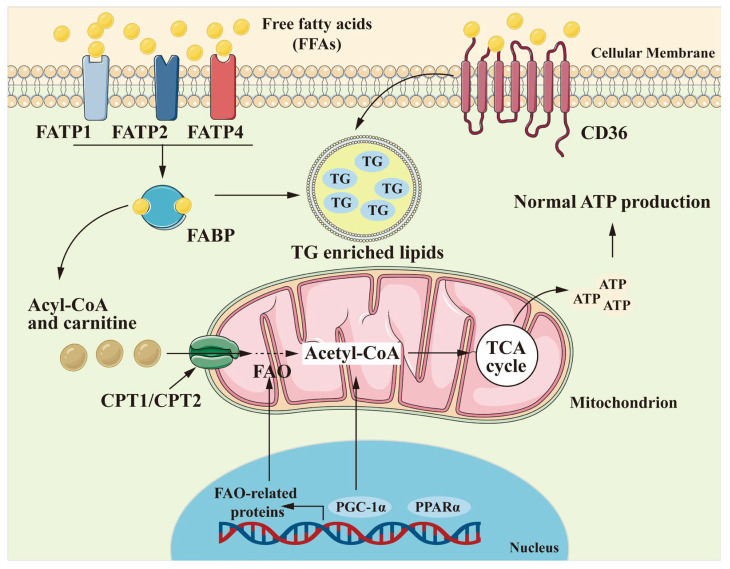
Key Mechanisms of Fatty Acid Metabolism in Renal Cells. Figure 2 depicts the primary pathways of fatty acid metabolism within renal cells. Triglycerides and cholesterol from the diet are broken down into free fatty acids (FFAs) and glycerol. FFAs enter cells via specific transport proteins: fatty acid transporter proteins (FATP1, FATP2, FATP4) and the scavenger receptor class B protein CD36. Within the cell, FFAs undergo fatty acid oxidation (FAO, also known as β-oxidation) or are stored in lipid droplets. The carnitine palmitoyltransferase enzymes CPT1 and CPT2 are crucial for transporting Acyl-CoA into the mitochondria, supporting ATP production. Mitochondrial biogenesis genes, particularly PGC-1α and PPARα, further enhance FAO-related protein expression and mitochondrial function. TG, triglycerides.

FFA uptake in the kidney is mediated by CD36, fatty acid transport proteins (FATPs), and fatty acid-binding proteins (FABPs) (Figure 2) [41,42,43,44]. These transporters facilitate the movement of FFAs across the plasma membrane into the cytoplasm, where they are either utilized for energy production or stored as triglycerides. CD36, an FFA translocase, plays a crucial role in the uptake of FFAs. It is widely expressed in various renal cells, including tubular epithelial cells, podocytes and mesangial cells [43]. Studies have shown that loss-of-function mutations in the *CD36* gene lead to abnormal plasma levels of FFAs and triglycerides in mice, underscoring its importance in lipid metabolism [45]. CD36 has also been linked to lipid accumulation in obesity-related glomerulopathy. For example, Yang et al. demonstrated that inflammation exacerbates kidney injury by activating the CD36 pathway in obese mice [46]. In conditions of diet-induced obesity or hyperlipidemia, oxidized low-density lipoprotein (oxLDL) or the cardiotonic steroid ouabain can activate CD36/Na^+^/K^+^-ATPase-dependent inflammatory pathways in proximal tubular cells (PTCs), contributing to chronic inflammation, oxidative stress, and fibrosis [47]. In podocytes, CD36-mediated uptake of palmitic acid triggers a dose-dependent increase in reactive oxygen species (ROS), mitochondrial membrane depolarization, ATP depletion, and activation of apoptotic pathways [48].

FATPs, encoded by the *Slc27* gene family, are another essential class of FFA transporters [41]. FATP2 (encoded by *slc27a2*), the predominant isoform in the kidney, is localized to the apical membrane of PTCs and plays a critical role in FFA uptake. A study by Khan et al. using Bodipy-labeled non-esterified fatty acids (NEFAs) demonstrated enhanced time- and concentration-dependent NEFA uptake in wild-type mice compared to *Slc27a2* knockout mice, which provides direct evidence that FATP2-mediated NEFA uptake in PTCs contributes to tubule atrophy and interstitial fibrosis [41]. FABPs belong to a superfamily of lipid-binding proteins with low molecular weight, which exist in various subtypes depending on tissue expression, including liver (L-FABP), heart (H-FABP), and adipocyte (A-FABP) isoforms [44]. Liver-type FABP (L-FABP or FABP1) is the predominant isoform in PTCs [49], which has been shown to promote lipid accumulation in obesity-related CKD. High-fat diet-induced reduction in the renal expression of *Fabp1* further emphasizes its importance in renal lipid uptake [50]. Together, CD36, FATPs, and FABPs orchestrate FFA uptake and metabolism in the kidney.

Cellular cholesterol homeostasis is another important component of kidney lipid metabolism. Cholesterol primarily enters renal cells through LDL, which bind to LDL receptors (LDLR) on the cell surface [26]. This LDL–LDLR complex is internalized via clathrin-mediated endocytosis, releasing free cholesterol into the cell. Proprotein convertase subtilisin/kexin type 9 (PCSK9) regulates this process by binding to LDLR and promoting its degradation in endosomes. By reducing LDLR levels, PCSK9 effectively limits cholesterol uptake [26]. In high-fat diet-fed mice, reduced circulating PCSK9 levels were found to promote CD36-dependent lipid accumulation in the kidneys, suggesting that PCSK9 may protect against lipid-induced kidney damage [51]. Interestingly, while CD36 is primarily known for facilitating FFA uptake, it also plays a role in cholesterol uptake [52]. Once inside the cell, free cholesterol is transported to various organelles by lipid-binding proteins such as Niemann-Pick C1 (NPC1) and NPC2. To prevent toxicity, excess cholesterol is esterified by acyl-CoA:cholesterol acyltransferase (ACAT) and stored in lipid droplets [26].

### 2.3. Lipogenesis in Obesity-Related Kidney Disease

Once inside the cell, FFAs undergo desaturation, elongation, and esterification processes. These activated FFAs are catalyzed by diacylglycerol acyltransferase (DGAT) and ACAT in the endoplasmic reticulum (ER) to synthesize neutral lipids, primarily triacylglycerols and sterol esters [53]. The neutral lipids initially form lens-like structures within the ER bilayer, gradually fusing into larger, more stable droplets or budding off to form nascent lipid droplets. Lipogenesis is primarily regulated by three key transcription factors: sterol regulatory element-binding proteins (SREBPs), peroxisome proliferator-activated receptor γ (PPARγ), and farnesoid X receptor (FXR).

SREBPs are a major regulator of FFAs and cholesterol synthesis, including SREBP-1 and SREBP-2 [54]. SREBP-1 serves as a powerful activator of a broad range of SREBP-responsive genes, driving the synthesis of cholesterol, fatty acids, and triglycerides. In comparison, SREBP-2 plays a more specialized role, primarily promoting the transcription of genes involved in cholesterol biosynthesis. SREBP-1, in particular, has gained attention for its role in promoting lipid synthesis in the context of obesity [55]. In obese individuals, SREBP-1 is upregulated, leading to increased expression of genes involved in FFAs and triglyceride synthesis [56]. This elevation in SREBP-1 activity drives triglyceride accumulation in renal cells, resulting in lipotoxicity and contributing to the progression of CKD [57,58]. The dysregulation of SREBP-1 in the kidney may occur through multiple mechanisms, including insulin resistance and the influence of inflammatory cytokines, commonly observed in obesity. Moreover, the crosstalk between SREBP-1 and other signaling pathways, such as the mTOR pathway, further complicates the regulatory network governing lipid metabolism in the kidneys [59].

PPARγ is primarily known for its role in regulating adipocyte differentiation and lipid storage [60]. It facilitates the conversion of excess FFAs into triglycerides, thereby preventing lipotoxicity [61]. However, increased PPARγ activity in renal cells has been linked to enhanced triglyceride accumulation, contributing to renal lipotoxicity [62]. Increased histone acetylation of PPARγ and SREBP-1 has been associated with heightened lipid accumulation in renal cells, contributing to the pathogenesis of CKD [63]. The transcription factor FXR primarily activated by bile acids—especially chenodeoxycholic acid, its most potent endogenous ligand—plays a protective role in metabolic and renal disorders. Studies have shown that FXR agonists confer renal protection in obesity-related CKD by downregulating SREBP-1 and upregulating PPARα, CPT1a, and PGC-1α [64]. FXR activation also effectively reduces kidney fibrosis, inflammation, and lipid accumulation through the AMPK/SIRT1/PGC-1α/SIRT3/ERRα signaling pathway while inhibiting ER stress, HIF signaling, and GLUT1 [65]. These mechanisms highlight FXR potential as a therapeutic target for mitigating kidney damage in diabetes and obesity.

### 2.4. FAO in Obesity-Related Kidney Disease

The kidneys require substantial ATP to sustain their daily functions, with distinct substrate preferences across regions reflecting varying energy demands. Glomerular cells primarily rely on glucose, while tubular cells, due to their high metabolic activity, favor fatty acid oxidation (FAO) as their main energy source [66]. Conversely, podocytes, endothelial cells, and mesangial cells in the glomeruli primarily depend on glycolysis but can shift to FAO as an alternative energy source under metabolic stress, such as during glucose depletion, to maintain cellular function [38]. FAO is a multi-step biochemical process that involves the transport of FFAs into the mitochondria, where they undergo β-oxidation to generate ATP [37,67,68]. However, reduced FAO and excessive lipid accumulation in these cells can lead to lipotoxicity, characterized by mitochondrial dysfunction, increased oxidative stress (OS), and ultimately cell death [69,70].

PPARα is predominantly expressed in renal tubular cells and plays a pivotal role in promoting FAO [71]. Upon activation by circulating FFAs, PPARα regulates the expression of genes encoding proteins involved in mitochondrial and peroxisomal FAO, thereby facilitating the clearance of excess lipids from renal tissues [72]. Importantly, recent studies suggest that the activation of PPARα may provide protective effects in obesity-related kidney injury by promoting the utilization of FFAs and attenuating inflammation [73]. So, the therapeutic potential of PPARα agonists, such as fenofibrate, in mitigating kidney injury highlights the significance of PPAR signaling pathways in maintaining lipid homeostasis within the kidney.

The carnitine palmitoyltransferase enzymes (CPT1 and CPT2) also play critical roles in the FAO process [74]. CPT1, located on the outer mitochondrial membrane, facilitates the conversion of FFAs into acylcarnitines, allowing for their transport into the mitochondrial matrix [68]. CPT2 then catalyzes the reverse reaction, converting acylcarnitines back into FFAs within the mitochondria, where they can be oxidized for energy [75,76]. Dysregulation of these key enzymes can lead to impaired FAO, resulting in excessive lipid accumulation within renal cells. In states of obesity or insulin resistance, alterations in the expression or activity of CPT1 and CPT2 may disrupt normal lipid metabolism, leading to reduced ATP production, mitochondrial dysfunction, and an increased susceptibility to cellular stress and apoptosis [77,78,79].

### 2.5. Lipid Export in Obesity-Related Kidney Disease

In the kidney, FAO is the primary mechanism for reducing lipid accumulation, with lipid export playing a relatively minor role [31]. Lipid export mainly relies on high-density lipoproteins (HDL). Chang et al. demonstrated that patients with higher HDL levels and lower HDL variability had a reduced risk of developing nephropathy, indirectly linking lipid export to kidney health [80]. However, further in vitro and in vivo studies are necessary to clarify the role of HDL in obesity-related nephropathy. For cholesterol, newly synthesized cholesterol is either esterified by SOAT1 or transported to the plasma membrane for efflux via the transporters ATP-binding cassette transporter A1 (ABCA1) and ABCG1 [26]. ABCA1 facilitates cholesterol and phospholipid efflux through an ATP-dependent mechanism. Podocyte-specific ABCA1 knockout in *ob/ob* mice exacerbated podocyte injury by promoting cardiolipin accumulation and mitochondrial dysfunction [81]. Additionally, TNF-α treatment downregulated the nuclear factor of activated T cells 1 (NFAT1)/ABCA1 axis, contributing to cholesterol-induced podocyte injury [82]. However, the role of ABCG1 in obesity-related nephropathy remains unclear and requires further investigation. The key regulators in lipid metabolism are summarized in Table 1.

## 3. Obesity-Induced Nephropathy: Cellular and Molecular Mechanisms

**Figure 3:**
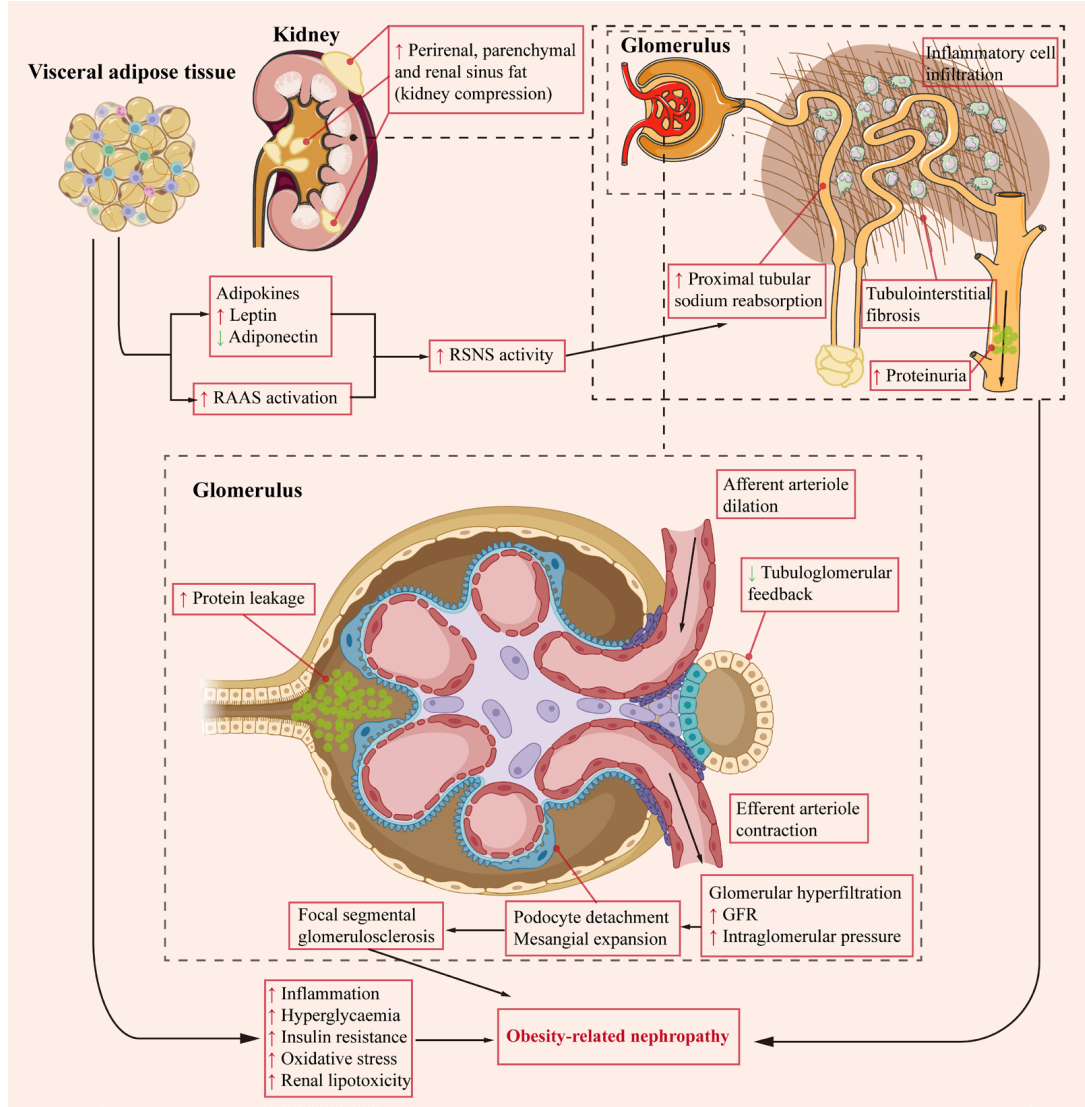
Mechanisms Underlying Obesity-Induced Nephropathy. Figure 3 illustrates the mechanisms contributing to obesity-related nephropathy. Hemodynamic changes, renin–angiotensin–aldosterone system (RAAS) activation, increased renal sympathetic nervous system (RSNS) activity, adipokine release from visceral adipose tissue, and physical compression by renal fat all play a role. Visceral adipose tissue releases angiotensin, aldosterone, and various adipokines, leading to RSNS activation and subsequent renal lipotoxicity. Increased proximal tubule reabsorption and RAAS activation diminish tubuloglomerular feedback, causing afferent arteriolar dilation, while angiotensin and aldosterone promote efferent arteriolar vasoconstriction. Together, these effects cause glomerular hyperfiltration, with a rise in glomerular filtration rate (GFR) and intraglomerular hypertension. Podocyte detachment and mesangial cell expansion contribute to focal segmental glomerulosclerosis. Lipid accumulation in renal cells further exacerbates tubular atrophy and interstitial fibrosis, accompanied by inflammation in the tubulointerstitial space. Collectively, these disruptions advance the development of obesity-related nephropathy.

Excessive fat accumulation, often associated with obesity, can extend beyond adipose tissue to vital organs, including the kidneys. This ectopic lipid deposition disrupts normal lipid homeostasis and cellular composition, leading to toxic lipid buildup and impaired kidney function. Obesity impacts renal function through various mechanisms, including intrarenal lipid accumulation, oxidative stress (OS), mitochondrial dysfunction, chronic inflammation, adipokine dysregulation, and activation of the renin–angiotensin–aldosterone system (RAAS) (Figure 3).

### 3.1. Lipid Deposition in the Kidney

#### 3.1.1. Increased Circulating FFAs

In obesity, there is a significant increase in circulating FFAs due to heightened lipolysis in adipose tissue [83]. This metabolic derangement is characterized by an imbalance in lipid homeostasis, where the release of FFAs from adipose tissues into the bloodstream exceeds the kidney’s ability to metabolize and utilize these lipids [84,85]. The accumulation of FFAs in the renal circulation can lead to an overload situation for renal tissues [16,86]. The kidneys, particularly the PTCs, are equipped to handle a certain level of FFAs; however, in obese individuals, the excess supply overwhelms their metabolic capacity. This excess of circulating FFAs leads to lipid overload within renal cells, subsequently resulting in the formation of lipid droplets [34,87]. These lipid droplets serve as reservoirs for excess lipids, attempting to buffer the toxic effects of excessive FFAs [88,89].

#### 3.1.2. Lipid Droplet Formation in Renal Cells

Lipid droplets, composed mainly of triglycerides and sterol esters, are dynamic organelles that play a critical role in lipid storage and metabolism within renal cells [90,91]. Under normal physiological conditions, lipid droplets help regulate cellular lipid levels and provide energy sources during periods of metabolic demand [92,93]. However, excessive lipid accumulation in renal cells, as observed in obesity, can lead to substantial pathological changes. Studies have indicated a strong correlation between the accumulation of lipid droplets in renal tissues and the progression of CKD in obese animals or patients [94,95]. When lipid storage exceeds the capacity of renal cells, it disrupts normal cellular functions, resulting in a state of lipotoxicity [57,96,97]. The excessive accumulation of lipid droplets can also interfere with cellular signaling pathways and exacerbate metabolic dysfunction, leading to cellular apoptosis and renal injury.

#### 3.1.3. Mechanisms of Lipotoxicity

Lipotoxicity is a significant concern in the context of obesity-related kidney disease. It arises when the excessive presence of lipids disrupts normal cellular functions, ultimately leading to cellular stress and death. The mechanisms underlying lipotoxicity are complex and multifactorial, involving mitochondrial dysfunction, ER stress, and the activation of pro-inflammatory pathways. Mitochondrial dysfunction, in particular, plays a pivotal role in lipotoxicity [31]. Excessive FFAs can impair mitochondrial function, leading to reduced ATP production and increased ROS generation [98]. Additionally, excessive FFAs may induce ER stress by overwhelming the protein-folding capacity of the ER, triggering the unfolded protein response [99]. This response, while initially protective, can result in apoptosis if the stress is prolonged or excessive. Therefore, the lipid-lowering strategy might be a potential therapy for obesity-related CKD.

### 3.2. OS and Lipid Peroxidation

OS results from an imbalance between ROS production and the endogenous antioxidant defenses. The primary source of ROS is oxidative phosphorylation (OXPHOS) in mitochondria. The endogenous antioxidant defense system includes enzymatic antioxidants such as superoxide dismutase (SOD), catalase, glutathione peroxidase, and thioredoxin, as well as non-enzymatic antioxidants like glutathione and vitamin E. Studies have shown that SIRT3 expression is downregulated in palmitic acid-stimulated PTCs, leading to reduced mitochondrial oxidative capacity and diminished SOD expression [100]. In palmitic acid-treated HK2 cells, ROS accumulation increased, while NRF2 protein expression decreased [101]. Similarly, Lee et al. demonstrated that palmitic acid treatment elevated ROS levels in mouse renal mesangial cells and podocytes [102]. PPARα also plays a crucial role in modulating OS through pathways such as AMPK/AKT/GSK3β and PI3K/AKT/FOXO3a [31]. As discussed in the treatment section, PPARα agonists like fenofibrate are used to enhance kidney function in obese patients. Consequently, lipid overload can lead to both glomerular and tubular damage via oxidative stress.

Lipid peroxidation products, such as malondialdehyde (MDA) and 4-hydroxynonenal (4-HNE), are generated during the oxidative degradation of polyunsaturated fatty acids in the presence of ROS [103]. These reactive aldehydes can modify cellular macromolecules, including proteins, lipids, and nucleic acids, leading to impaired cellular function and contributing to the progression of renal injury [104,105]. The accumulation of lipid peroxidation products in the kidneys can trigger inflammatory responses, promote apoptosis, and stimulate fibrosis, further contributing to the progression of CKD [53]. The detrimental effects of these lipid peroxidation products are compounded by their ability to activate pro-inflammatory pathways, creating a vicious cycle of inflammation and tissue damage.

### 3.3. Mitochondrial Dysfunction in Lipid Overload

Mitochondria are essential for kidney energy metabolism [106]. However, in obesity, excessive lipid accumulation within renal cells can lead to mitochondrial dysfunction, characterized by impaired oxidative phosphorylation and decreased ATP production. The overloading of FFAs can result in mitochondrial structural changes and the opening of the mitochondrial permeability transition pore, leading to cell death [107,108]. The mitochondria-targeting drug SS-31 has been shown to preserve mitochondrial membrane potential, effectively mitigating lipotoxicity, podocyte and endothelial cell loss, glomerulosclerosis, and inflammation in high-fat diet fed mice [109]. These findings highlight mitochondrial function as a potential treatment target for obesity-related kidney disease. In this section, we focus on mitochondrial DNA (mtDNA) damage and impaired mitochondrial biogenesis.

#### 3.3.1. mtDNA Damage

In obesity, excessive production of ROS leads to OS, which can cause oxidative damage to mtDNA [110]. This damage impairs mitochondrial function, leading to diminished energy production and compromised lipid metabolism in renal cells [111]. The accumulation of damaged mtDNA further exacerbates mitochondrial dysfunction, creating a vicious cycle of lipid overload and cellular injury. Moreover, mtDNA damage has been linked to the activation of pro-apoptotic pathways, leading to renal cell apoptosis and subsequent loss of renal function [112]. Recent studies have highlighted the role of mitochondrial integrity in maintaining kidney health [113], suggesting that strategies aimed at protecting mtDNA from oxidative damage could hold therapeutic potential in obesity-related kidney diseases.

#### 3.3.2. Impaired Mitochondrial Biogenesis

Mitochondrial biogenesis is a critical process for maintaining mitochondrial mass and function, regulated by several key factors, including PPARγ coactivator 1-alpha (PGC-1α) [114]. PGC-1α plays a vital role in promoting the expression of genes involved in mitochondrial biogenesis and FAO. However, in the context of obesity, reduced PGC-1α expression is often observed, leading to diminished mitochondrial biogenesis and impaired mitochondrial function in renal cells [115]. The decrease in mitochondrial mass and function due to impaired biogenesis contributes to energy deficits and increased susceptibility to lipotoxicity in renal cells [111,116].

### 3.4. Inflammation and Fibrosis in Obesity-Related CKD

#### 3.4.1. Inflammatory Cytokines and Pathways

Obesity is often characterized by a state of chronic low-grade inflammation, with the increased production of pro-inflammatory cytokines such as tumor necrosis factor-alpha (TNF-α), interleukin-6 (IL-6), and monocyte chemoattractant protein-1 (MCP-1) [117,118]. These cytokines play crucial roles in promoting renal inflammation and fibrosis, thereby exacerbating the progression of CKD. Inflammatory cytokines can recruit immune cells to the renal interstitium, further perpetuating the inflammatory milieu and leading to tubular damage [119]. This chronic inflammatory state can result in the activation of fibroblasts and the deposition of extracellular matrix components, ultimately contributing to renal fibrosis and loss of function [120,121]. For instance, two meta-analyses have identified genetic polymorphisms in inflammatory cytokines, such as TGF-β1, IL-4, IL-6, and IL-10, as significant contributors to the susceptibility to CKD and diabetic nephropathy [122,123]. Additionally, the NLRP3 inflammasome has been implicated in the pathogenesis of obesity-related CKD. In podocytes, high-fat diet-induced activation of the NLRP3 inflammasome disrupts autophagy, leading to the accumulation of phospholipids in dysfunctional lysosomes and subsequent renal injury [124]. NLRP3 deficiency or pharmacological inhibition in obese mice has been shown to reduce renal lipid droplet accumulation, inflammation, fibrosis, and albuminuria, highlighting the potential therapeutic value of targeting this pathway [125,126]. The relationship between lipid accumulation and inflammation in the kidney is intricate and reciprocal. Lipid overload in tubular cells activates various inflammatory pathways, which exacerbate renal injury. FFAs can stimulate the expression of pro-inflammatory cytokine in renal tubular cells, which in turn attract inflammatory cells and perpetuate a cycle of inflammation and cellular damage. Chronic inflammation in obesity can lead to sustained activation of inflammatory pathways, such as nuclear factor-kappa B (NF-κB) signaling, which further contributes to the production of inflammatory mediators [127]. This prolonged inflammatory state can promote renal fibrosis and accelerate the progression to CKD. Therefore, targeting the inflammatory response alongside lipid metabolism could be a promising strategy for mitigating renal injury in obese individuals [128].

#### 3.4.2. Epithelial-Mesenchymal Transition (EMT)

The interplay between lipid-induced damage in tubular cells and the inflammatory environment can trigger epithelial–mesenchymal transition (EMT), a process in which epithelial cells lose their characteristic markers and acquire a fibroblast-like phenotype [129,130]. This transition is driven by various signaling pathways activated during renal injury, including TGF-β and Wnt/β-catenin signaling pathways. EMT is a critical contributor to the fibrotic response in the kidneys, leading to increased matrix production and subsequent deterioration of renal architecture and function [131]. The contribution of EMT to fibrosis in obesity-related CKD emphasizes the need for targeted therapeutic strategies aimed at mitigating lipid accumulation, OS, and inflammatory responses to preserve kidney function. In conclusion, obesity alters kidney function through direct and indirect pathways.

## 4. Lipid Metabolism Disorders and Kidney Pathology

### 4.1. Glomerular Lipotoxicity and Glomerulosclerosis

#### 4.1.1. Podocyte Dysfunction in Obesity

Podocytes possess unique structural features, including foot processes that interdigitate to form slit diaphragms, essential for maintaining selective permeability [132]. In the context of obesity, increased lipid levels in circulation can lead to lipotoxicity, which significantly impacts podocyte function. Lipotoxicity manifests in podocytes as morphological changes, including effacement of foot processes, which disrupts the integrity of the filtration barrier. This effacement is often associated with a decrease in the expression of nephrin and podocin, key proteins integral to the podocyte’s structural framework [133]. Consequently, the loss of podocyte integrity leads to increased protein permeability and the development of proteinuria, a hallmark of glomerular dysfunction [57,134]. The impairment of podocytes is not merely a consequence of lipid overload; it is also influenced by inflammatory processes that occur in obesity. Thus, the interplay between lipid accumulation and inflammation creates a vicious cycle that enhances glomerular damage and the progression of kidney disease [135].

#### 4.1.2. Role of Mesangial Cells to Glomerulosclerosis

Mesangial cells, located between the glomerular capillaries, provide structural support and regulate glomerular filtration [136,137]. In obesity, these cells can also undergo lipid accumulation, leading to adverse effects on glomerular function. Mesangial cells can internalize FFAs through specific transporters, resulting in increased lipid droplet formation. This accumulation is not merely a passive response but can actively contribute to glomerulosclerosis. Lipid-laden mesangial cells promote glomerular fibrosis by producing excessive extracellular matrix (ECM) components, such as collagen and fibronectin [138]. This process leads to thickening of the glomerular basement membrane and expansion of the mesangial matrix, both of which impair glomerular filtration. Additionally, mesangial cell activation can be stimulated by pro-inflammatory cytokines, further promoting a fibrotic response within the glomerulus. The resultant glomerulosclerosis is a significant pathway leading to progressive renal dysfunction, ultimately contributing to the development of CKD.

### 4.2. Tubulointerstitial Injury

The tubules of the nephron are essential for the reabsorption of water, electrolytes, and other solutes. In obesity, excessive lipid accumulation in tubular cells can lead to detrimental effects on renal function. The accumulation of FFAs in PTCs induces cellular stress, characterized by mitochondrial dysfunction, ER stress, and inflammation. This loss of functional tubular cells can lead to impaired solute reabsorption and diminished renal concentrating ability [139]. Moreover, the accumulation of lipids can exacerbate tubular atrophy and interstitial fibrosis, which is a critical component of renal scarring and progression to CKD. This process of tubular injury is often accompanied by an inflammatory response in the tubulointerstitial space. The infiltration of immune cells, particularly macrophages, contributes to the local inflammatory milieu and perpetuates renal damage [140]. Additionally, tubular injury can lead to the release of pro-fibrotic factors that promote ECM deposition, further contributing to renal scarring and dysfunction.

### 4.3. Progression to Chronic Kidney Disease

Dysregulated lipid metabolism is a significant contributor to the progression of CKD, particularly in the context of obesity. Clinically, individuals with obesity-related CKD often present with various complications, including increased proteinuria, reduced estimated glomerular filtration rate (eGFR), and elevated levels of inflammatory markers [14,141,142]. The presence of proteinuria is particularly concerning, as it is a strong predictor of progressive kidney disease and cardiovascular events. Moreover, alterations in lipid profiles, such as elevated circulating FFAs and dyslipidemia, are commonly observed in patients with obesity-related kidney disease. Thus, monitoring lipid metabolism and implementing strategies to normalize lipid levels may have critical implications for managing CKD progression in obese individuals [143].

## 5. Therapeutic Strategies in Obesity-Related CKD

Therapeutic strategies for obesity-related CKD focus on lipid modulation, antidiabetic agents, and lifestyle interventions (Table 2). Statins and fibrates improve renal outcomes by reducing lipid levels, inflammation, and proteinuria. SGLT2 inhibitors and AMPK activators, such as metformin, enhance fatty acid oxidation and mitigate kidney damage beyond glycemic control. Lifestyle modifications, including physical activity and dietary changes like omega-3 supplementation and reduced saturated fat intake, improve renal function and metabolic health. Emerging therapies targeting lipid synthesis pathways, such as SREBP-1 inhibitors, hold promise but require further research. Holistic approaches integrating pharmacological and lifestyle interventions are essential for optimal CKD management.

### 5.1. Pharmacological Approaches Targeting Lipid Profiles

#### 5.1.1. Statins and Renal Protection

Statins, a class of drugs primarily used to lower LDL cholesterol levels, have gained attention for their potential renal protective effects, particularly in patients with obesity-related CKD. Statins exert their beneficial effects not only through lipid-lowering mechanisms but also via anti-inflammatory and pleiotropic actions [144]. Studies have demonstrated that statins can improve renal outcomes in patients with CKD by reducing proteinuria and slowing the progression of renal dysfunction [145,146]. For instance, Study of Heart and Renal Protection (SHARP) clinical trials have indicated that statin therapy is associated with a significant reduction in the risk of end-stage renal disease (ESRD) in diabetic patients with CKD [147]. The mechanisms underlying these protective effects include the modulation of the RAAS, reduction of OS, and improvement of endothelial function [148]. By lowering inflammatory markers such as C-reactive protein (CRP), statins may alleviate chronic inflammation, a key driver of renal injury in obesity. Furthermore, the use of statins has been shown to have a favorable impact on cardiovascular outcomes in CKD patients, addressing the comorbidities frequently observed in individuals with obesity and kidney disease [147]. However, it is essential to monitor for potential adverse effects, such as muscle toxicity and liver function abnormalities, to ensure the safety and efficacy of statin therapy in this population.

#### 5.1.2. Fibrates: Modulating Lipid Profiles

Fibrates, including fenofibrate and gemfibrozil, are another class of lipid-modulating agents that enhance FAO and lower triglyceride levels. They activate PPARα, a key regulator of lipid metabolism, leading to increased lipoprotein lipase activity and enhanced clearance of triglyceride-rich lipoproteins [149]. Clinical evidence suggests that fibrates may improve renal function and reduce proteinuria in patients with dyslipidemia and CKD [150]. In particular, fenofibrate has been investigated for its reno-protective properties in the context of type 2 diabetes, a common comorbidity in obese patients [151]. Studies have shown that fenofibrate can significantly reduce urinary albumin excretion, indicating improved glomerular permeability and protection against nephropathy. Moreover, the combination of statins and fibrates has been explored for its potential synergistic effects on lipid profiles and renal outcomes [152]. However, careful consideration is required to manage the risk of adverse events, such as myopathy and increased liver enzymes, when using these agents in conjunction.

#### 5.1.3. SREBP-1 Inhibitors: Targeting Lipid Synthesis Pathways

As discussed above, SREBP-1 is a transcription factor that regulates lipid synthesis and is upregulated in obesity. Inhibiting SREBP-1 could be a promising strategy to reduce lipid synthesis and prevent lipid overload in renal cells. Researchers are investigating the potential of SREBP-1 inhibitors as therapeutic agents to ameliorate the impact of dysregulated lipid metabolism on kidney health [56]. By targeting the pathways involved in lipid metabolism, SREBP-1 inhibitors may help mitigate diabetic nephropathy progression by reducing lipotoxicity and improving overall renal function [153]. However, clinical trials for SREBP-1 inhibitors are not yet available. Further research is needed to explore the efficacy and safety of these agents in clinical settings, but they represent an exciting avenue for future therapeutic interventions.

### 5.2. Antidiabetic Agents Targeting Obesity-Related CKD

#### 5.2.1. SGLT2 Inhibitors: Dapagliflozin and Empagliflozin

Sodium–glucose cotransporter 2 (SGLT2) inhibitors, including dapagliflozin and empagliflozin, have emerged as effective therapeutic agents for managing obesity-related CKD, particularly in patients with type 2 diabetes. These agents work by promoting glycosuria, leading to osmotic diuresis and a reduction in blood glucose levels. Recent clinical trials have highlighted the renal protective effects of SGLT2 inhibitors, demonstrating significant reductions in proteinuria and slowing of CKD progression [154,155]. The EMPA-REG OUTCOME trial and the DAPA-CKD trial provided robust evidence for the efficacy of empagliflozin and dapagliflozin, respectively, in improving renal outcomes in patients with established CKD and cardiovascular disease [154,155]. The renal protective mechanisms of SGLT2 inhibitors extend beyond glycemic control [156,157]. These agents improve renal outcomes by reducing hyperfiltration in the glomeruli, lowering intraglomerular pressure, and modulating inflammation [158]. By enhancing FAO in renal tubular cells, SGLT2 inhibitors help mitigate lipid-induced renal injury, thus improving overall renal health. The reduction of uric acid levels and improvement of endothelial function also play a role in mitigating renal injury [159], highlighting the multifaceted mechanisms through which SGLT2 inhibitors exert their benefits in obesity-related CKD. Although not approved for weight loss, SGLT2 inhibitors can lead to a 1–3 kg reduction in body weight [160]. Additionally, they may benefit the kidneys by reducing fat accumulation and lowering blood pressure [161]. These effects suggest that SGLT2 inhibitors have significant potential to alleviate kidney damage associated with obesity.

#### 5.2.2. AMPK Activators: AICAR and Metformin

Adenosine monophosphate-activated protein kinase (AMPK) is a crucial energy sensor that regulates cellular metabolism. Activating AMPK can improve mitochondrial biogenesis, enhance FAO, and inhibit lipid synthesis, making it a promising therapeutic target for obesity-related CKD [162]. Reagents such as 5-aminoimidazole-4-carboxamide ribonucleoside (AICAR), a pharmacologic activator of AMPK and metformin have shown potential in preclinical models by improving lipid accumulation, mitochondrial function, and reducing oxidative stress in cisplatin-induced acute kidney injury [163] and high-fat diet-induced mice [164]. Metformin, a widely used medication for type 2 diabetes, has demonstrated protective effects in the kidneys, independent of its glucose-lowering effects [165,166]. The reno-protective effects of metformin are primarily attributed to its ability to reduce lipid accumulation and fibrosis through the activation of AMPK signaling. However, studies have shown that metformin can still reduce inflammatory markers and promote an antioxidant response in kidney tissue, even in mice lacking AMPK, suggesting that it may also exert its protective effects through AMPK-independent pathways [167]. Metformin is known to induce significant weight loss in individuals with or without diabetes [168], although the exact mechanisms underlying this effect remain a subject of debate. Furthermore, Brosnahan et al. assessed the safety and tolerability of metformin in patients with autosomal dominant polycystic kidney disease [169]. While participants in the metformin group experienced more adverse symptoms, primarily gastrointestinal issues and diarrhea, these effects were generally resolved either spontaneously or after reducing the metformin dose [169]. Despite these findings, there is still a lack of clinical trials exploring the use of AMPK activators or metformin in the context of obesity-related chronic kidney disease.

### 5.3. Lifestyle and Dietary Interventions

#### 5.3.1. Importance of Weight Loss in CKD Management

Weight loss is a crucial component of managing obesity-related CKD, as it can lead to significant improvements in renal function and overall health [28]. Studies have shown that caloric restriction and physical activity can result in decreased proteinuria, improved GFR, and reduced risk of cardiovascular events in obese patients with CKD [170,171]. A systematic review analyzing six primarily lifestyle intervention studies found that a hypocaloric diet, either alone or combined with exercise, was generally effective in reducing weight, blood pressure, and proteinuria, while preserving eGFR during short-term follow-up [172]. In addition, in the Look AHEAD (Action for Health in Diabetes) trial, overweight and obese participants who received intensive lifestyle interventions, including caloric restriction and exercise, experienced a 31% reduction in the incidence of very-high-risk CKD compared to those receiving standard diabetes support education [173]. Weight loss interventions can enhance insulin sensitivity, reduce systemic inflammation, and improve lipid profiles, all of which contribute to better kidney health [174]. Implementing structured weight loss programs, including dietary counseling and physical activity regimens, is essential for optimizing outcomes in this patient population.

#### 5.3.2. Physical Activity

Exercise therapy offers significant cardiometabolic benefits and improves renal function, in addition to promoting weight loss. In a meta-analysis of 13 randomized controlled trials, non-dialysis CKD patients who engaged in exercise therapy showed significant improvements in eGFR, along with reductions in body mass index (BMI) and blood pressure compared to controls [175]. Both aerobic exercise and resistance training have been shown to have anti-obesity effects, influencing inflammation, oxidative stress, and the progression of CKD [176]. A 4-month clinical study by Ikizler et al. on aerobic exercise in patients with severe CKD demonstrated that aerobic exercise significantly reduced inflammatory responses and improved metabolic health in those with moderate-to-severe CKD [177].

#### 5.3.3. Dietary Modifications: Omega-3 Fatty Acids and Reduced Saturated Fats

Dietary modifications play a significant role in managing lipid metabolism and promoting renal health. Increasing the intake of omega-3 fatty acids and reducing saturated fat consumption can have positive effects on renal outcomes [178]. Omega-3 fatty acids, found in fatty fish and flaxseed, possess anti-inflammatory properties and have been shown to improve renal function in patients with CKD [179,180]. Omega-3 supplementation may lead to reduced proteinuria and improved kidney function, potentially through mechanisms involving the modulation of inflammatory pathways and lipid metabolism. Conversely, a diet high in saturated fats can exacerbate inflammation and oxidative stress, negatively impacting kidney health. Adopting a heart-healthy diet, such as the Mediterranean diet, which emphasizes whole grains, fruits, vegetables, lean proteins, and healthy fats, can provide significant benefits for individuals with obesity-related CKD [178]. In the Dietary Intervention Randomized Controlled Trial (DIRECT), Tirosh et al. followed patients with obesity and CKD for a median of two years. They concluded that three dietary approaches—low-fat, Mediterranean, and low-carbohydrate restricted-calorie diets—had similar effects on renal function, as evidenced by improvements in eGFR and reductions in albuminuria [178]. These dietary patterns help normalize lipid profiles and enhance overall health outcomes in this vulnerable population. Recently, the low-calorie ketogenic diet (LCKD) has gained popularity as an effective tool for weight loss and the treatment of obesity-related diseases [181]. However, LCKD is generally considered contraindicated in CKD patients due to the increased risk of metabolic acidosis and nephrolithiasis [182]. Therefore, ketogenic diets for CKD patients should be plant-based and followed under strict supervision by a multidisciplinary team.

In conclusion, therapeutic strategies for obesity-related CKD offer diverse benefits but also face limitations. Statins effectively lower LDL cholesterol, reduce inflammation, and slow CKD progression, though risks like muscle toxicity and liver dysfunction require monitoring. SGLT2 inhibitors provide renal protection beyond glycemic control and modest weight loss but are not yet approved for non-diabetic CKD. AMPK activators, such as metformin, show potential in reducing lipid accumulation and oxidative stress but need more research for CKD-specific outcomes. Lifestyle interventions improve renal function and metabolic health, although adherence and individual variability can limit long-term success. Emerging therapies like SREBP-1 inhibitors are promising, but remain in early development stages, necessitating further investigation into efficacy and safety.

## 6. Future Directions

### 6.1. Unanswered Questions in Lipid Metabolism Research

Despite the growing body of evidence linking lipid metabolism to the progression of CKD in obese individuals, several critical questions remain unanswered. One of the primary areas of interest is the elucidation of specific molecular pathways through which dysregulated lipid metabolism contributes to renal injury [26,27]. For instance, while it is known that lipid accumulation in renal cells leads to lipotoxicity and oxidative stress, the precise molecular interactions and signaling cascades involved in these processes require further investigation. Additionally, the role of various lipid species—such as FFAs, ceramides, and lipoproteins—in modulating renal function is not yet fully understood. Emerging research suggests that certain lipid metabolites may have distinct effects on renal cell types, leading to differential outcomes in terms of inflammation and fibrosis. For example, the role of sphingolipid metabolism in podocyte injury and glomerulosclerosis remains an area ripe for exploration [183]. Future studies should also investigate the impact of lifestyle interventions, such as diet and exercise, on lipid metabolism and kidney health. Understanding how modifications in lifestyle can influence lipid profiles and subsequent renal outcomes will be crucial in developing comprehensive management strategies for obesity-related CKD. Longitudinal studies and clinical trials are needed to compare the effects of lifestyle interventions on renal function in obesity-related kidney disease. The mechanisms through which these interventions confer benefits can be investigated by monitoring changes in relevant metabolites (including but not limited to lipid metabolites) in blood or urine before and after the interventions. Furthermore, animal experiments could provide deeper insights into the underlying mechanisms. By implementing various dietary interventions, molecular changes in the kidneys can be directly examined, and target-specific interventions can help establish causal relationships between dietary modifications and their molecular targets. By integrating findings from molecular, clinical, and epidemiological studies, researchers can create a more nuanced understanding of the relationships between obesity, lipid metabolism, and kidney health.

### 6.2. Development of Biomarkers for Early Detection

Identifying biomarkers associated with lipid metabolism disorders is essential for the early detection and management of CKD in obese patients. Current clinical practices often rely on conventional markers of kidney function, such as serum creatinine and urinary albumin excretion [184]. However, these markers may not adequately capture the early stages of kidney dysfunction, particularly in the context of dysregulated lipid metabolism. Biomarkers such as circulating FFAs, lipoprotein profiles, and lipid peroxidation products can provide valuable insights into the pathophysiological changes occurring in the kidneys. For instance, elevated levels of specific FFAs, such as palmitic acid, have been linked to increased renal lipotoxicity and inflammation [185]. Additionally, alterations in lipoprotein profiles, particularly an increase in small, dense LDL particles, can signify a higher risk for cardiovascular and renal complications [186]. Lipid peroxidation products, such as MDA and 4-HNE, serve as markers of oxidative stress and can indicate ongoing cellular damage in renal tissues.

Advancements in metabolomics and lipidomics are paving the way for the discovery of novel biomarkers that reflect lipid profiles and metabolic disturbances associated with renal injury [187]. By employing high-throughput techniques, researchers can analyze the comprehensive lipidome of patients to identify specific lipid signatures linked to CKD progression. These biomarkers could facilitate the early diagnosis of kidney dysfunction, allowing for timely interventions to prevent or mitigate disease progression. Furthermore, integrating proteomic analyses with genetic and epigenetic profiling may enhance our understanding of individual susceptibility to CKD in the context of obesity [188]. By integrating kidney biopsy transcriptome data and urine protein analysis with baseline and longitudinal clinical follow-up data, Ju et al. identified EGF (encoding the protein epidermal growth factor) in the kidney as positively correlated with eGFR [189]. The researchers then explored whether urine EGF (uEGF) could predict the decline in kidney filtration function over time. Their findings demonstrated that the uEGF-to-creatinine ratio is associated with an increased risk of CKD [190]. This integrative approach could lead to the identification of predictive biomarkers that not only reflect lipid metabolism but also account for genetic predispositions and environmental influences. Such biomarkers could prove invaluable in clinical practice, guiding personalized treatment strategies for patients at risk of developing obesity-related kidney disease.

### 6.3. Personalized Medicine Approaches in Treating Obesity-Related CKD

As the understanding of obesity-related kidney disease evolves, the need for personalized medicine approaches becomes increasingly evident. The heterogeneity observed in obesity-related CKD underscores the importance of tailoring therapeutic strategies to the individual patient. Factors such as genetic background, lipid metabolic profiles, and comorbidities play a critical role in determining treatment responses and outcomes. Personalized medicine approaches may involve the use of lipid metabolic profiling to stratify patients based on their specific lipid abnormalities [191]. By categorizing patients into distinct subgroups, clinicians can design targeted interventions that address the unique metabolic derangements present in each individual. For instance, patients with high circulating levels of specific FFAs may benefit from therapies aimed at enhancing FAO, while those with elevated triglycerides may require interventions focused on lipid-lowering strategies.

Additionally, personalized approaches can extend beyond pharmacological interventions to encompass lifestyle modifications. Tailoring dietary recommendations and exercise regimens based on individual lipid metabolic profiles may enhance treatment efficacy and improve patient adherence. Integrating technology, such as wearable devices that monitor physical activity and dietary intake, could further support personalized management plans and empower patients to take an active role in their health [192]. In conclusion, advancing our understanding of lipid metabolism’s role in obesity-related CKD requires a multifaceted research approach that addresses existing gaps in knowledge and translates findings into clinical practice. By focusing on the molecular mechanisms underlying lipid dysregulation, developing innovative biomarkers for early detection, and embracing personalized medicine strategies, we can improve outcomes for individuals affected by obesity-related kidney disease. The convergence of metabolic research, clinical innovation, and patient-centered care holds the potential to transform the landscape of CKD management in the context of obesity, ultimately leading to better health outcomes and enhanced quality of life for affected individuals.

## 7. Conclusions

Obesity-induced lipid metabolism disorders are critical factors in the development and progression of CKD. The dysregulation of lipid homeostasis not only contributes to renal injury but also exacerbates systemic inflammation and oxidative stress, leading to a vicious cycle of kidney dysfunction. Targeting lipid metabolism through various therapeutic strategies, including pharmacological agents and lifestyle modifications, holds promise for preventing or slowing CKD progression. Emerging therapies such as SGLT2 inhibitors and AMPK activators are demonstrating protective effects on renal function in obese patients. Furthermore, lifestyle interventions, including weight loss and dietary modifications, play a pivotal role in managing lipid levels and improving overall kidney health. Ongoing research is essential to fully elucidate the underlying mechanisms driving lipid-induced renal damage, to develop effective treatments, and to optimize patient outcomes. 

## Figures and Tables

**Table 1:** Key molecular regulators and their roles in renal lipid metabolism.

Regulator	Role in Lipid Metabolism	Effect on Kidney Health
CD36	FFA and cholesterol uptake	Increases FFA levels, promotes lipotoxicity
FATPs	FFA uptake	Increases FFA levels, promotes lipotoxicity
FABPs	FFA uptake	Increases FFA levels, promotes lipotoxicity
LDLR	Cholesterol uptake	Increases free cholesterol levels
PCSK9	Regulates cholesterol uptake	Reduces LDLR levels, limits cholesterol uptake
SREBP-1	Drives lipid synthesis	Increases triglyceride levels, promotes lipotoxicity
PPARγ	Regulates lipid storage and adipogenesis	Contributes to lipid accumulation in obesity
FXR	Regulates lipid synthesis	Protects against lipotoxity, downregulates SREBP-1 expression
PPARα	Promotes FAO	Protects against lipid-induced renal injury
CPT1/CPT2	Promotes FAO	Protects against lipid-induced renal injury
HDL	Lipid export	Protects against lipid-induced renal injury
ABCA1	Cholesterol export	Facilitates cholesterol and phospholipid efflux

FATPs, Fatty Acid Transport Proteins; FABPs, Fatty Acid-Binding Proteins; LDLR, Low-Density Lipoprotein Receptors; PCSK9, Proprotein Convertase Subtilisin/Kexin Type 9; SREBP-1, Sterol Regulatory Element-Binding Protein 1; PPAR, Peroxisome Proliferator-Activated Receptor; FXR, Farnesoid X Receptor; FAO, Fatty Acid Oxidation; CPT, Carnitine Palmitoyltransferase Enzymes; HDL, High-Density Lipoproteins; ABCA1, ATP-Binding Cassette Transporter A1.

**Table 2:** Pharmacological approaches and mechanisms of action.

Drug Class	Example(s)	Mechanism of Action	Effect on Kidney Health
Statins	Atorvastatin, Rosuvastatin	Lowers LDL cholesterol, reduces inflammation	Reduces proteinuria, slows CKD progression
Fibrates	Fenofibrate, Gemfibrozil	Enhances FAO, reduces triglycerides	Decreases lipid accumulation, reduces proteinuria
SGLT2 Inhibitors	Dapagliflozin, Empagliflozin	Lowers blood glucose, reduces hyperfiltration	Reduces proteinuria, slows CKD progression
AMPK Activators	Metformin, AICAR	Enhances FAO, decreases lipid synthesis	Improves mitochondrial function, reduces oxidative stress

LDL, Low-Density Lipoprotein; CKD, Chronic Kidney Disease; FAO, Fatty Acid Oxidation; SGLT2, Sodium–Glucose Cotransporter 2; AMPK, AMP-Activated Protein Kinase; AICAR, 5-Aminoimidazole-4-carboxamide Ribonucleotide.

## Data Availability

Individual data can be found in the referenced manuscripts.
